# The timing of female genital mutilation and the role of contralateral palpal insertions in the spider *Cyclosa argenteoalba*

**DOI:** 10.1098/rsos.170860

**Published:** 2017-11-22

**Authors:** Kensuke Nakata

**Affiliations:** Kyoto Women's University, Kitahiyoshi-cho 35, Higashiyama-ku, Kyoto 605-8501, Japan

**Keywords:** female genital mutilation, mating behaviour, paternity, spider, sexual conflict

## Abstract

Some male spiders exhibit female genital mutilation behaviour (FGM) by removing the female genital appendage (scape) to control the mating frequency of females. Female spiders have two, i.e. right and left, genital openings connected with separate spermathecae into which males transfer sperm successively using one pedipalp (secondary genitalia) at a time. Thus, males must complete at least two palpal insertions to fill both spermathecae, before FGM. The present study examined whether (i) scape removal is only associated with the second palpal insertion (one-action hypothesis) or (ii) two contralateral palpal insertions facilitate FGM, with each insertion cutting the basal part of the scape halfway (two-actions hypothesis). Experiments in which females were replaced after a male had made the first insertion did not support the one-action hypothesis, because scapes remained intact after the newly introduced virgin females received their first palpal insertion, which was the second insertion by the males. In comparison, mating experiments using two half-eunuchs (i.e. one of the palps of each male had been manually removed, forcing them to fill female spermatheca on one side only) supported the two-actions hypothesis. FGM was more frequent in females that received two contralateral palpal insertions than in females that received ipsilateral insertions.

## 1. Background

In the majority of taxa, a female often mates with multiple males [1,2]. A female that has more than one reproductive partner might benefit from higher fecundity and improved genetic quality and/or diversity of her offspring [3,4]. However, this action reduces male fitness; thus, to secure paternity, males have developed various strategies to inhibit females from mating with multiple males, including guarding females [5], injecting chemical substances to lower female re-mating tendency [6] and mating plugs [7].

Another way to prevent females from mating multiply is through female genital mutilation (FGM) by males. For example, FGM has been described in two orb-web spiders, *Cyclosa argenteoalba* [8] and *Larinia jeskovi* [9]. In these species, virgin females have a small projection, called a scape, on their external genitalia (epigynum) [10]. The scape is essential for successful mating. The male must ‘clasp’ the scape with its genitalia (pedipalp) to position itself appropriately for palpal insertion [11]. In FGM, the scape is removed from the epigynum during mating, preventing the success of subsequent genital coupling. Thus, mutilated females cannot mate with additional males. FGM is a powerful way in which males can inhibit females from mating with multiple partners, and thus ensure full male paternity. As a result, FGM contrasts with other ways to secure paternity that are more costly to males in terms of energy expenditure and mating opportunity. For instance, mate guarding might reduce the glycogen reserves of males in stream-dwelling isopods [12]. Some male spiders form mating plugs by breaking their pedipalps and leaving breakages in the copulatory openings of females [13,14]. Male *Nephilengys malabarensis* also detach their entire pedipalp, which continues sending sperm into a female. Such an emasculated and, thus, lightened male can guard the female from other males more efficiently [15–17]. Other males sacrifice themselves as food to females in copula, to send more sperm while being eaten [18]. All these males lose all their future mating opportunities. Male garter snakes adjust the size of mating plugs according to the size of the females [19], suggesting that the production of these plugs is costly. Nevertheless, males often fail to monopolize their partnered female [20–22]; thus, monandry is relatively rare [23]. Thus, it is important to understand FGM to elucidate the evolution of monandry/polyandry and evolutionary outcomes of male–male competition over paternity and male–female conflict over the number of times a female copulates.

*Cyclosa argenteoalba* is a diurnal spider that occurs in Japan, Korea and China [24]. This species builds vertical orb webs. The male spider has two pedipalps (i.e. right and left), while the females of entelegyne spiders, including *C. argenteoalba*, have two copulatory openings, each of which is connected to separate spermatheca. On mating, a male makes a mating thread and sends courtship signals by tapping and jerking the thread with its legs. When a female accepts courtship, the male inserts one of its pedipalps into one of the genital openings of the female. Thus, at least two palpal insertions are required to fill both spermatheca with sperm, which is considered to lower the risk of insufficient sperm being available at oviposition [25]. In fact, *C. argenteoalba* males insert their pedipalps twice in a single mating bout, using each pedipalp successively (i.e. one pedipalp first, followed by the other). When the insertion successfully ends, the pair separates, and the male repeats its courtship behaviour to make the second insertion using the other pedipalp. The scape typically remains on the epigynum during the first insertion; however, it is absent after the second insertion [8]. This process is logical because if FGM occurred at the first insertion, males could not complete the second insertion, and the second spermatheca would remain empty.

Here, we examine if selection acts on *C. argenteoalba* males to implement FGM after the second insertion in order to maximize paternity success. This question will give us invaluable insights into the evolutionary process and selective mechanisms of FGM. Namely we test two hypotheses. The one-action hypothesis predicts that spiders implement no actions to mutilate the scape during the first insertion, with scape-removal behaviour only being associated with the second insertion. Alternatively, the two-actions hypothesis predicts that a male destroys only one side (right or left) of the scape at the first insertion. Once both insertions (right and left) are complete, the destruction of the scapes on both sides facilitates successful FGM. The rationale for this hypothesis comes from a phenomenon recorded in *L. jeskovi*, in which a claw-like sclerite part of the pedipalp (called tegular apophysis) seems to ‘slash’ the side of the basal part of the scape of females halfway during copulation [9]. Thus, this study aimed to examine which of these two hypotheses is valid in *C. argenteoalba*.

## 2. Material and methods

I collected both adult and subadult female *C. argenteoalba* from Shimamoto, Osaka and from Nagaoka-Kyo, Japan between 2014 and 2015. All females were found on moulting webs, indicating that they had just completed the final moult (adult) or were ready for the final moult (subadult) [26]. Subadult females were allowed to moult into adults. By following this procedure, I ensure that all adult females were virgins prior to the experiment. Females were released into the observation area (9 m × 2 m) where they built their webs. Individual spiders were identified by variation in the abdominal markings [27] and the location of their webs. The observation area was not enclosed, but was surrounded by residential buildings. The nearest natural habitat of *C. argenteoalba* was more than 1 km away, with no obvious signs of immigration (e.g. additional webs by immigrating spiders or unknown males residing in the female web) observed during the study. Adult males, for which the mating history was not known, were also collected from the same sites and were maintained in separate vials with wet cotton.

## 3. Female-exchange experiment

Ten pairs of virgin females and males were subjected to staged mating. When a male successfully inserted one of its pedipalps and completed sperm transfer (I could not determine whether the right or left pedipalp was used, due to the small size of the spiders and short duration of insertion), females were removed from the web. After the male resumed courtship, a different virgin female was introduced to the hub of the experimental web from a nearby web. Soon, the second virgin female was aware of the courting male, and accepted its first, but the male's second, insertion. The male did not exhibit any further mating behaviour, and left the web. I inspected the second female under the microscope to check for the presence of the scape.

## 4. Half-eunuch experiment

Adult males were anaesthetized with CO_2_, and the tip of one of their pedipalps was cut off with fine scissors under the microscope. Preliminary observation confirmed that manipulated males could only make one insertion during a single mating event, indicating that the operation successfully disabled the manipulated pedipalp. Which pedipalp was removed (right or left) for each male was randomly determined. Thirty-two experimental females were assigned to four groups. Females from the first group were coupled with a male that had an intact right pedipalp (termed right-hand male, hereafter). After receiving one palpal insertion from the first male, the female was coupled with another male with an intact left pedipalp (hereafter, left-hand male), and received the second insertion. Females from the second group were coupled with a left-hand male first, and then with a right-hand male. The females in the third and fourth groups were coupled with two right-hand and left-hand males, respectively. Owing to issues that arose during the handling of spiders, the sample size was not balanced (*N* = 7, 8, 9 and 8 in groups 1, 2, 3 and 4, respectively). As a result, one right-hand male was used twice as the second male for females in the second and the third groups. Removing the data of this male from the analysis did not affect the qualitative aspect of the results. All other males were used only once. After a female received two insertions, the presence of the scape was inspected under the microscope. The mutilation rate was similar between the first and the second groups, and between the third and fourth groups. As a result, data from the first and second groups were pooled as the contralateral insertion group, and the data from the third and fourth groups as the ipsilateral group. The difference in the frequency of mutilation between the contralateral and ipsilateral insertion groups was examined by Fisher's exact probability test.

## 5. Results

In the female exchange experiment, none of the second females lost their scapes. In the half-eunuch experiment, 10 of the 15 females from the contralateral insertion group and four of the 17 females from the ipsilateral insertion group lost their scapes. This difference was statistically significant (*p* = 0.031).

## 6. Discussion

The results of the female-exchange experiments showed that when females received only one palpal insertion, no FGM occurred, even when it was the second insertion for males. In comparison, the half-eunuch experiments showed that the incidence of FGM was significantly higher in females that received two contralateral insertions than in females that received ipsilateral insertions. Thus, results supported the two-actions hypothesis over the one-action hypothesis. The results of the present study suggest that, during palpal insertion, *C. argenteoalba* males slash the side of the scape, similarly to that recorded in *L. jeskovi* [9]; thus, slashing both right and left sides of the scape facilitates FGM.

In the half-eunuch experiment, some females from the ipsilateral insertion group lost their scapes. A previous study showed that *C. argenteoalba* females lost their scapes only after receiving one palpal insertion, although at a low rate (only two in 44 mutilations observed) [8]. Thus, cutting both sides of the scape might not always be required for FGM. As argued in the study on *L. jeskovi* [9], additional action, such as twisting off the scape, might be involved in successful mutilation. In the typical mating ritual of *C. argenteoalba*, a copulating male and female hang from a mating thread by the third and the fourth legs, and their bodies often rotate along the vertical line, passing through the point of genital conjunction immediately after the timing of insertion. This body rotation might generate a twisting force, which, if strong enough, might cause genital mutilation, even when only one side of the scape is cut. The necessity of additional action is logical because if the scape was removed at the exact timing of the second slashing, which is considered to occur when the pedipalp clasps the scape, subsequent palpal insertion would fail.

FGM seems to require two insertions. Two-actions FGM might be essential for the efficiency of securing paternity because when a male has made only a single palpal insertion, failing to insert the palp a second time, the scape remains attached to the female. This event might be expected when a female cannibalizes a male after the first palpal insertion or when the mating sequence was interrupted after the first insertion by a disturbance, such as sudden change in weather, the destruction of the web or when two males simultaneously court the same female. If males only mutilated the scape during the second insertion, as expected by the one-action hypothesis, the scape of a female that had received only one insertion would remain undamaged. Consequently, a second male could make two successful palpal insertions. Assuming that two males inject a similar amount of sperm per insertion, the sperm of the first male would represent one-third of the sperm mixture. By contrast, if the male cut the scape halfway during the first insertion, as expected by the two-actions hypothesis, the opportunity for a second male to make two palpal insertions would be lowered. Assuming that males exhibit no preference in the use of their pedipalps, the second male might insert its pedipalp into a contralateral genital opening. Then, the scape would detach, preventing the second insertion. Without cryptic female choice, in this instance, the paternity share of the first male would be raised to half. Thus, cutting the scape halfway is considered beneficial to secure paternity, even if this action alone does not lead to mutilation.

FGM appears to be related to sexual conflict. Under sexual conflict, males often enforce costs on their mating-partner females and females exhibit counter-adaptations [28,29], and theoretical study revealed that FGM may evolve even when females suffer fecundity costs [30]. In some insects, males damage the internal genitalia of females in copula and females resist behaviourally to this harmful copulation [31,32]. In *C. argenteoalba*, females do not seemingly exhibit any behaviour to resist mutilation [8]. Nevertheless, an arms race on the efficiency of mutilation might occur between males and females, in the form of antagonistic genital coevolution [33]. In *Cyclosa*, some species other than *C. argenteoalba* do not exhibit FGM (personal observation). Comparison of the morphology of genitalia, especially the basal part of the scape in females and tegular apophysis in males, among species that do and do not exhibit FGM might provide information about the evolution of genitalia, including details on the mechanism of mutilation.

## Data accessibility

All data are available in the text.
